# Knowledge translation for public health in low- and middle- income countries: a critical interpretive synthesis

**DOI:** 10.1186/s41256-018-0084-9

**Published:** 2018-10-22

**Authors:** Catherine Malla, Paul Aylward, Paul Ward

**Affiliations:** College of Medicine and Public Health, Flinders University, Sturt Road, Bedford Park, Adelaide, South Australia 5042 Australia

**Keywords:** Critical interpretive synthesis, Knowledge translation, Low- and middle- income countries, Public health

## Abstract

**Background:**

Effective knowledge translation allows the optimisation of access to and utilisation of research knowledge in order to inform and enhance public health policy and practice. In low- and middle- income countries, there are substantial complexities that affect the way in which research can be utilised for public health action. This review attempts to draw out concepts in the literature that contribute to defining some of the complexities and contextual factors that influence knowledge translation for public health in low- and middle- income countries.

**Methods:**

A Critical Interpretive Synthesis was undertaken, a method of analysis which allows a critical review of a wide range of heterogeneous evidence, through incorporating systematic review methods with qualitative enquiry techniques. A search for peer-reviewed articles published between 2000 and 2016 on the topic of knowledge translation for public health in low- and middle – income countries was carried out, and 85 articles were reviewed and analysed using this method.

**Results:**

Four main concepts were identified: 1) tension between ‘global’ and ‘local’ health research, 2) complexities in creating and accessing evidence, 3) contextualising knowledge translation strategies for low- and middle- income countries, and 4) the unique role of non-government organisations in the knowledge translation process.

**Conclusion:**

This method of review has enabled the identification of key concepts that may inform practice or further research in the field of knowledge translation in low- and middle- income countries.

**Electronic supplementary material:**

The online version of this article (10.1186/s41256-018-0084-9) contains supplementary material, which is available to authorized users.

## Background

There is international recognition that accessing and using health research is a vital component of improving health and reducing health inequities [1]. However, there are substantial complexities that affect the way in which public health research is utilised for action in low and middle income countries (LMICs) [2, 3]. The “unacceptable gap between unprecedented knowledge about diseases and their control, and implementation of that knowledge” described by Sanders et al. ([3], p.758), highlights the need for better utilisation of research evidence, particularly in LMICs where the burden of poor health and healthy inequity is high. Analysing the factors that contribute to this gap may contribute to enabling solutions for better utilisation of research in LMICs [3].

Knowledge Translation (KT) – which goes by a host of different terms [4, 5] - describes the process of using evidence to make decisions and create action [6]. A commonly used definition of KT is the Canadian Institutes of Health Research’s:

“*A dynamic and iterative process that includes synthesis, dissemination, exchange and ethically-sound application of knowledge to improve [health]…. provide more effective health services and products and strengthen the health care system…. within a complex system of interactions between researchers and users”* [7].

KT provides a mechanism by which the inequities in public health outcomes for LMICs can be reduced [8]. Research on KT processes can provide an opportunity to examine how KT can better contribute to reducing these global health inequities [9].

In the context of public health in LMICs, there are substantial complexities that affect the process of KT. Some of these relate to the realities of living in resource-poor settings, such as low levels of infrastructure and a lack of financial, technical and skilled human resources [10, 11]. There are also complexities that are borne out of existing structural inequities, such as the historical influence of high-income countries in the field of global health and development, and questions of who and what shape the research agenda, and how this research is used [3, 12–15].

The following is a review of the literature on KT for public health practice and policy in LMICs, focusing on the contextual factors that influence the access to and utilisation of research evidence. This review takes the form of a critical interpretive synthesis (CIS) as described by Dixon-Woods et al. [16] and attempts to draw out themes in the literature that contribute to defining the current state of public health KT in LMICs. It is not intended to be a comprehensive review of what works in KT in LMICs or to describe KT processes in LMICs in detail, nor relate KT in LMICs to existing theories and models of KT, some of which has been carried out elsewhere [17–19]. Rather, this review attempts to examine the research on KT processes in LMICs and draw out and analyse some of the complexities and contextual factors that influence KT in this setting.

## Methods

### Literature review type

The CIS method of analysis is a review type that incorporates systematic review methods with qualitative enquiry techniques to enable the synthesis of a range of types of evidence. From this analysis, ‘synthetic constructs’ are generated, in order to draw together themes to form new concepts or theories [16, 20, 21]. The CIS method was chosen for this review because it allows for the synthesis of a large and diverse body of literature, followed by the development of concepts through an interpretive analysis [16]. The literature on KT in LMICs is indeed large and diverse, and prior to the analysis, it was not known what the key concepts would be, rather they were allowed to developed during the synthesis, as per the CIS method [16]. The CIS process begins with developing a ‘compass question’ to initially guide the literature search and analysis, and then an iterative process is followed that enables the question to be modified in response to search results [16]. The compass question that this review began with was “*How is evidence accessed and used by policy-makers and public health practitioners for improving public health in low- and middle- income countries?*”

### Search strategy

The search strategy was kept intentionally wide-ranging, adopting two broad search terms: “research utilisation” (and related terms) and “LMICs” (and related terms). Consideration was given to narrowing the search through including the search term “public health”, however it was felt that this could potentially omit sources that were public health-related but did not specifically use this term. Despite yielding a high number of source articles, this broad search was adopted to maximise the likelihood of capturing all relevant publications. Additional articles were purposively sampled in order to fill conceptual gaps during the course of analysis (in line with the CIS method) such as general papers on research utilisation/knowledge transfer that would form part of the introduction, discussion and the theoretical basis of the literature review. The entire search strategy can be found in Additional File 1. Databases searched were Medline, PubMed, CINAHL, Scopus, Web of Science, Google Scholar, Cochrane Library and PsycINFO. The database searches were limited by English language, humans, and between the years 2000–2016. Table 1 shows the number of articles that were retrieved from each electronic database.

**Table 1:** Electronic database search results

Database	Articles retrieved
Medline	3806
PubMED	930
PsycINFO	3145
CINAHL	3211
Cochrane	304
Web of Science	1163
Scopus	944
Google Scholar	240
TOTAL	13,743

### Search results

**Fig. 1:**
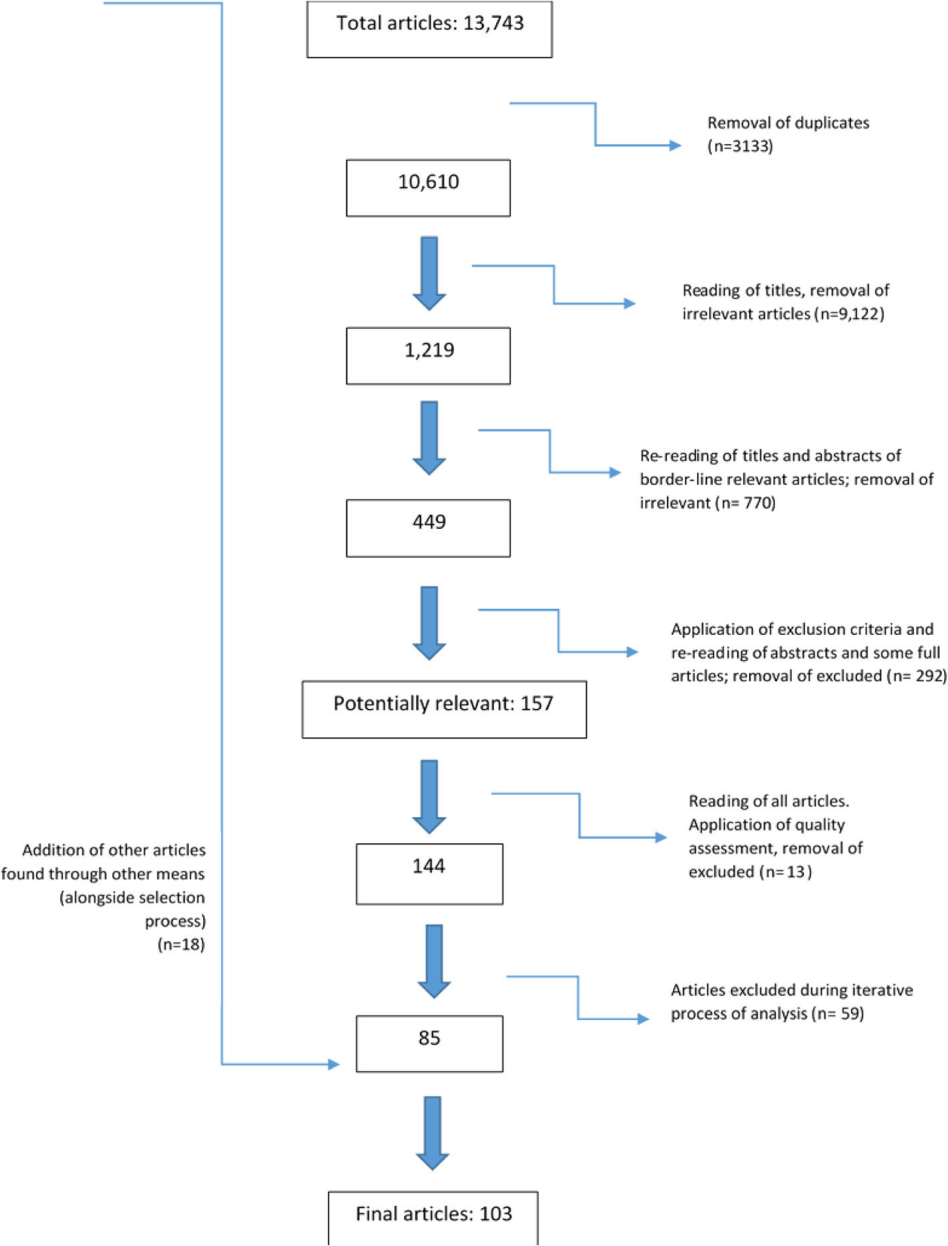
“PRISMA” diagram outlining process of electronic database and other searching (as per [22])

A “PRISMA” [22] flow diagram (Fig. 1) summarises the process of article selection. A total of 13,743 articles were retrieved from the electronic database search, and after removal of duplicates the list numbered 10,610. After reading titles and removal of irrelevant articles, 1219 remained. Upon further reading of titles and abstracts of articles that were border-line relevant, a further 770 were removed, leaving 449. At this stage, exclusion criteria were applied using title and abstract, and where required, the full article. Developing exclusion criteria at this stage was necessary as the articles displayed such a wide range of setting and contexts for KT, and the number of articles for the review needed to be kept at a manageable size. The exclusion criteria that were developed aimed to ensure that the articles chosen were most relevant to the compass question.

Articles that focused on the following were excluded:

1. Clinical practice rather than broader public health
2. Knowledge translation activities directed at clinicians
3. Knowledge translation activities directed at community project beneficiaries
4. Not strictly related to low- and middle-income countries
5. Suggestions that an innovation/trial should be taken up, rather than an actual process
6. Laboratory/biomedical processes
7. Non-health related issues
8. Translating policy into action
9. Protocol papers

After applying the exclusion criteria, 157 potentially relevant articles remained which were each critically examined, and details entered into a data extraction table (Additional File 2). The table was developed to capture key concepts from the articles that would allow them to be categorised into themes that could be further developed later into the ‘synthetic constructs’ required for a CIS. The data extraction table also categorised the articles by type of article, methods and key findings.

At this stage, the articles were also assessed by the first author for quality, utilising a quality assessment tool, drawing on Fane et al.’s [21] use of Attree’s grading system from A-C [23] whereby an A grading was for primary research or review with high relevance to the compass question, B was primary research or review with less relevance, and C was an opinion/commentary, description of programs, or an article that provided background information only. Thirteen papers were excluded at this point. During the process of the CIS analysis of the remaining papers, additional papers (*n* = 59) were excluded, as it became apparent that they were no longer relevant enough to provide insight into the compass question.

Some articles that were not results of the original search were added during the process of analysis (*n* = 18). These were sourced by scanning the reference lists of the chosen articles for any potentially relevant articles and checking the citations of selected articles using Google Scholar if it was felt that this would provide further insights on a particular concept. Other articles were sourced through Google Scholar in order to fill conceptual gaps.

Types of articles included in the review included primary research (quantitative, qualitative and mixed methods), systematic and scoping reviews, program descriptions and conceptual papers/opinion pieces. Given the large amount of relevant material identified, grey literature was not searched, however specific documents were purposively sourced to capture definitions or the position of a global body such as the World Health Organisation (WHO) (*n* = 4). Table 2 is a summary of the characteristics of the articles, by year and type and method of searching (format modified from Moat, Lavis [20]).

**Table 2:** Characteristics of articles reviewed

Characteristic	Number	
	Searches	Purposive
Year published		
pre- 2000	0	1
2000–2005	4	4
2006	2	0
2007	3	0
2008	1	1
2009	5	1
2010	9	3
2011	9	3
2012	9	2
2013	6	1
2014	16	1
2015	18	0
2016	3	1
TOTAL	85	18
Article type		
Conceptual and opinion/commentary	18	5
Primary Research:		
*Qualitative*	47	2
*Quantitative*	4	1
*Mixed methods*	4	1
Reviews	9	5
Description of program	3	0
Grey literature	0	4
TOTAL	85	18

As each article was read, details were added into the data extraction table (Additional File 2).

### Analysis

This review used a CIS approach, and therefore the analysis was an iterative process, with some papers being excluded and added during the analysis process itself, as described above. The analytic approach of a CIS involves the development of ‘synthetic constructs’ and then a ‘synthesising argument’ [16]. A ‘synthetic construct’ interprets and transforms the underlying evidence into a new conceptual form; and then these ‘synthetic constructs’ are integrated together to form a ‘synthesising argument’ that interprets the evidence as a whole [16, 20].

The data extraction table was used to draw out the key findings in each paper that related to the compass question. Using NVivo, key findings were coded into general themes which were developed during the process of reading the articles, of which there were 12 at the end of the process.

The groups of key findings coded into each general theme were then re-examined, with the original article consulted again if further clarification was required. Each article within a theme was then re-summarised into one paragraph (some articles had paragraphs in more than one theme), and then using these paragraphs, each theme was summarised into 4–5 key points (using only a few words of a phrase for each key point). All of these key points were written onto one sheet of paper, and from here the synthetic constructs were developed. During the process of writing the synthetic constructs, the synthesising argument was constructed. Additional File 3 shows which articles were aligned with each synthetic concept, as well as additional articles that were purposefully sampled to fill conceptual gaps.

## Results

Four synthetic constructs were developed through the analysis of these articles. These include (1) the tension between global and local health research, (2) complexities in creating and accessing evidence, (3) contextualising strategies for KT in LMICs, and (4) the unique role of NGOs in the KT process.

### Tension between ‘global’ and ‘local’ health research

Tension between the concepts of ‘global’ and ‘local’ is woven throughout the literature describing KT in LMICs. The distinction is made between ‘global’ evidence and ‘local’ evidence – ‘global’ being research on global level health issues or involving global organisations in defining, funding or carrying out research in LMICs; and ‘local’ being research that is produced at the national or sub-national level by local agencies in LMICs [13, 14, 24–27]. Burchett [28] describes a similar distinction made by public health stakeholders in Ghana between ‘big’ research (tending to be on national or international scale public health issues) and ‘small’ research (carried out at the local level, including operational research, evaluations or pilot projects, designed and controlled by local programme managers).

There can be a complex relationship between global and local evidence. Global-level evidence brings an international lens to certain issues such as non-communicable diseases and globalisation, as well as potential links to the latest international thinking and resources to carry out high quality research and provide in-country capacity building [29–31]. However, the importance of local evidence in the development of public health programs is being increasingly recognised, as it ensures that local priorities are recognised, and that research agendas are relevant for the country’s policy context and are more connected to realities at the country level [13, 26, 29, 30, 32]. The literature describes some programs that have not sufficiently taken on board local-level evidence, to their detriment [14, 25, 30, 33].

Partnerships between international and local organisations have been established as a way to link the production and application of global evidence with local contextual evidence. Strategies such as employing local researchers, directly funding local institutions, building collaborative networks, funding joint initiatives and building research capacity have been employed within such partnerships [24, 31, 34–36]. Some of these partnerships have prioritised and facilitated two-way learning where both partners learn and benefit [24, 31, 35], challenging the “paradigm of uni-directional problem solving” ([24], p.ii54).

However, there is concern that global-local partnerships can be characterised by power imbalances or even exploitation [13, 25, 37, 38]. This can mean that research priorities are set by the global partner, resulting in research agendas that have little relevance to local contexts and circumstance, and potential erosion of a country’s own research capacity [13, 26, 36, 38]. Although research partnerships may utilise local researchers or organisations, these can be inequitable relationships, where the local partners are in subordinate positions, on the “peripheries” ([13], p.1794, [15]), contributing to “historical inequity in the conduct and access to research” ([15], p.25). Sometimes LMICs are under pressure to undertake certain research as a precondition to receiving funding or loans [36, 37]. Cáceres and Mendoza [13] call for an increase in scrutiny on the growing number of global research collaborations, due to the intricate “political, institutional, economic and cultural variables” ([13], p.1792) that are involved in research in LMICs.

‘Global’ or ‘local’ status forms part of the assessment of the trustworthiness of evidence by public health decision-makers in LMICs, with policymakers acutely aware of the differences in these types of evidence [17, 34, 39, 40]. For some decision-makers, global research has a lower value due to its perceived lack of relevance [28, 40, 41] whereas local research is believed to be able to provide ‘hands-on’ evidence and hence has higher value [13, 32, 34, 40, 42]. On the other hand, local research can sometimes be seen to be of low quality due to the lack of skills of local researchers, or because it doesn’t provide the conceptual evidence that global research can sometimes provide [41]. Some stakeholders prefer a mixture of the two where one type of evidence can support the other [32, 39, 43]. Trust is important in valuing different types of research - if the researcher, institution or provider of knowledge can be trusted, the evidence is seen to be of higher value [32, 44–46]. Local researchers (those not from international organisations) may be considered trustworthy, depending on their reputation and level of authority [40, 44], however well-known institutions such as the WHO are considered intrinsically trustworthy in some instances [39, 44]. There is heavy reliance on the trustworthiness of the providers of evidence, as many decision-makers don’t have the capacity to critically analyse the research themselves or to be confident in their analysis of a situation [40, 47]. Knowledge brokers can play a key role as providers of evidence, as people or organisations whose role is to facilitate and mediate between researchers and decision-makers, adapting the research to the local context to reduce barriers to understanding between the two [5, 48, 49].

Two forms of evidence that are highly valued across many decision-making settings are local routine data collection and operational research, both forms of ‘small’ research. These types of data are seen as particularly useful, and often essential, by local stakeholders for several reasons: their ability to provide local context to policy and practice [28, 46, 50, 51]; the fact that they can be collected by local researchers, practitioners and other non-academic figures [52]; and the fact that most of the time the agenda for this data collection was set locally, not at a global level [28].

A wide variety of types of evidence is used to inform policy and practice in LMICs [28, 43, 46, 53, 54], ranging from formal studies and government reports, to “anything that is done to understand a situation” ([28], p.22). Evidence may be valued or defined by its “relevance, applicability and generalizability to a specific context” rather than necessarily its quality ([54], p.79), and can hold very different meanings depending on the way it is presented, and for which audience [46, 55]. For example, experience and discretionary judgement was the main source of evidence used in updating essential medicines guidelines in Tanzania, as opposed to scientific evidence such as cost-effectiveness studies [56]. Similarly, local burden of disease studies were preferred by countries making decisions on vaccines, rather than global data [39]. The potential of qualitative research evidence to broaden the evidence base in terms of providing context and explanations for quantitative findings in LMICs is discussed briefly in the literature [28, 57].

The country in which research is conducted can have an impact on its interpretation and dissemination possibilities. Research produced from low-income countries is less likely to be published than that by a researcher from a high-income country, reducing its dissemination potential [58–60].

### Complexities in creating and accessing evidence

Conducting public health research in some LMICs is limited by weak resource infrastructure and limited institutional capacity. These limitations include little or no government investment for research, low levels of training and skills for researchers, poor academic environments where researchers may work in isolation or combine research with clinical caseloads, limited or no peer review systems, and limited access to research tools such as analysis software [10, 27, 38, 41, 61]. These limitations contribute to the lack of a research culture sometimes found in LMICs, leaving countries open to “research imperialism” ([12], S4) where external agendas influence research [12, 13, 15, 25, 32]. They can also lead to limited incentives to carry out research that is policy-related [53] or to incorporate KT as part of the research process [62, 63]. Reluctance of local researchers to share data or research results can arise, due to uncertainties about who might be requesting data and why, concerns about data being misrepresented, and the fear of someone else publishing results without permission or appropriately acknowledging the original researcher [60, 64, 65]. Limited understanding of ‘Western’ approaches to research can also affect applications for research funding, resulting in a possible unfair advantage to researchers from high income countries [13, 28]. These patterns highlight the “balance of prevailing global power, perspectives and interests” ([2], p.1631) in accessing and utilising research evidence.

A globally-defined research agenda has meant that important areas of research have not been necessarily prioritised in LMICs [13], such as research on the social determinants of health [13, 61, 66], non-communicable diseases, urbanisation and health inequities [67], however this is changing rapidly [68]. Health systems research, another neglected area of public health research in LMICs [69], has tended not to be a priority of ‘big’ research, but can be beyond the scope of ‘small’ research [14, 28, 30, 70].

In some LMICs large amounts of data are collected in the form of national health surveys, program monitoring data and operational research [12, 50, 51, 71], creating a reservoir of potential ‘valuable’ local evidence for decision-making. However, the use of this data is limited by low levels of capacity to analyse it for dissemination for policy and practice [12, 53, 72] and issues of data quality [30, 41, 53, 73], hence the description “data rich, information poor” ([12], p.S4). Policymakers lament the difficulty in accessing relevant research findings that are of high quality and in digestible formats and they often have limited skills in interpreting evidence [30, 41, 53, 73]. They struggle with being provided an uneven mix of evidence from different sources that is difficult to evaluate, and with being able to ask the right questions for good policymaking [41, 73]. There is a role here for international agencies in supporting operational research and analysis, prompting a call for international support for LMICs to use their operational research data [72].

The unavailability of electronic databases for accessing research evidence due to cost and infrastructure is a significant impediment to its use for public health for some places in LMICs [17, 74, 75] . Some advances have been made in this area, such as the establishment of Hinari [76, 77] and similar programs [52, 75, 78] which are platforms that provide access to scientific literature for little or no cost to health knowledge users in LMICs. These schemes have provided much greater access to online health journals, however there are still some limitations relating to the dissemination of some of these programs, and the provision of practical training in their use [74, 79, 80]. Additionally, infrastructure issues such as inadequate hardware, poor Internet connections, and unreliable electricity serve as major impediments to the access and use of evidence [30, 44, 73, 81].

### Contextualising strategies for KT in LMICs

Utilisation of research findings to influence public health policymaking is complex [82] and this is compounded by the many other factors aside from research evidence that influence public health policy [19, 83]. In LMICs there are particular factors that add to this complexity, including the issues relating to power structures and capacity discussed above. It is important that KT strategies used in LMICs are contextualised for the cultural, political and economic decision-making context [17, 18, 38, 48].

There are many KT models described in the literature [84], however most have been developed in high-income countries and therefore may not be applicable in the context of LMICs [37, 63]. In general, there is a lack of awareness, knowledge and clarity of KT techniques in LMICs [17, 37, 62]. Despite this, there have been attempts to trial and utilise specific KT techniques in LMIC settings, often via a partnership approach. The literature describes a number of these techniques, including: systematic reviews [10], rapid response mechanisms [47], evidence briefs and deliberative dialogues [85, 86], KT platforms and formal knowledge networks [17, 87–90], integrated KT [91], the use of knowledge brokers [5, 48, 49] and social knowledge management [92]. Common features for success across these techniques include a strong training or capacity building element, ensuring the cultural, political and economic context is taken into account, and encouraging a collaborative approach across sectors and between researchers and decision-makers.

Factors that facilitate KT in LMICs have been reviewed by Orem et al. [17], who found the most significant factors to be institutional strengthening for KT, the characteristics of the research itself, and partnerships between researchers and policymakers. Building capacity for KT in LMICs requires institutional strengthening of both research and policymaking systems in order to promote a greater use of evidence in policymaking [13, 17, 37, 50, 53, 93, 94]. Existing capacity strengthening in these areas is sometimes ad hoc [53] or targeted at individuals rather than at an organisational level [27, 36, 94]. Institutional strengthening in policy-making systems requires resources for infrastructure as well as legitimacy and regulatory support [53, 95]. It requires technical capacity in critical research skills, knowledge management and in leading KT processes, stemming from a good understanding of the organisation’s capacity for research use in the first place [50, 93, 95].

The influence of partnerships between researchers and policy-makers provide clear advantage to KT, including both formal and informal knowledge networks and personal relationships [45, 54, 89, 95, 96]. Institutional platforms that allow researchers, policymakers and other stakeholders to engage with each other increases appreciation of each other’s processes and challenges [36, 87, 88, 95]. Such platforms may take the form of formal networks (including virtual networks), events, websites, or be a separate entity with an office [87, 88]. Longer-term links between institutions enables a continuous, rather than ad hoc, exchange of information, strengthening capacity of both the suppliers and the users of evidence [50, 53].

A number of strategies have been suggested for optimising the adoption of research findings by policymakers. A review of research characteristics that improve uptake of findings by policymakers [17] found the most effective to be timely, high quality, contextualised evidence that provides economically viable recommendations for policy options, preferably provided by local researchers with high credibility. One way in which research with these characteristics can be produced is through engaging in user-driven research agendas, which result from collaborations between researchers and decision makers [33, 40, 44, 69, 97]. Research that appeals to a political agenda can have a significant effect, such as in Nepal where data on the household cost of a birth attendant presented a novel perspective to health planners and influenced decision-making on maternal and child health policy [42].

Contextualising KT for LMICs can be aided by monitoring and evaluating KT processes, however the lack of systems for doing this in LMICs was noted in this literature [18, 26, 62, 88, 93, 98], and more widely [6]. The development of robust evaluation frameworks, systems and instruments for KT in LMICs is required for assessing the outcomes and impact of KT activities on changes in behaviour of decision-makers and the structural impact on health systems [18, 62, 88, 99]. Some examples of evaluations of KT strategies were found in the literature, however these were few [88, 98, 100].

### The unique role of NGOs in the KT processes

Non-government organisations (NGOs) in LMICs are in a potentially unique position to be involved in KT, and the literature found that they often carry out KT-related activities as part of their day-to-day activities. NGOs are generally known for their connections to the realities of what is happening at the community level, ability to mobilise communities, and their role in representing and advocating for the vulnerable [52, 93, 101–103], so are well situated to play a role in utilising evidence that promotes equity. NGOs have involvement with a range of KT processes in LMICs including managing, synthesising and utilising knowledge for practice and advocacy, disseminating findings, acting as knowledge brokers, implementing research findings, working with partners, advocating with policy makers to implement evidence, and having input into policymaking [17, 24, 38, 52, 93, 95, 98, 101–108].

NGOs in LMICs can have influence over the research process through being involved in research priority setting, resource mobilisation for research, promoting and advocating for relevant research, partnering with international agencies to ensure research relevance and effectiveness and conducting operational research [46, 52, 101–103, 108]. Having NGOs involved in research has been shown to increase the use of research findings in contributing to social development and health equity [103], can increase the role of community in research, and increase the effectiveness of their advocacy efforts [108]. Formal partnerships between NGOs in LMICs and researchers in global health are increasing, and one framework for navigating successful partnerships is described by Olivier et al. [102].

Despite this wide range of roles played by NGOs in KT, there has been little exploration of further capabilities of NGOs in this space in LMICs [3, 93, 95]. NGOs are important stakeholders in the field of public health and may often have potential capacity for a key role in public health KT [103, 107, 109, 110]. Supporting local NGOs to better generate and use research evidence has been suggested as an important way to improve KT generally [108–110].

## Discussion

Within the field of public health in general, there are few systems and structures in place to support the promotion and facilitation of KT strategies [82, 111]. In LMICs, structural inequities and limited resources have added barriers to the utilisation of evidence. This review highlights some of the complexities specific to LMICs that can be considered when developing KT strategies in these contexts. The findings from this review show that KT is influenced by both the nature of knowledge and the context under which these processes occur, and therefore that there is value in identifying and placing importance on these factors. Influencing or acting on such contextual factors may enable “conceptual and strategic ways to bring about changes in knowledge and understanding, or shifts in perception, attitudes and beliefs” ([112], p.189).

This review has identified four ‘synthetic constructs’ that attempt to interpret some of the research in the area of KT within LMICs, in order to provide a response to the original compass question: “*How is evidence accessed and used by policy-makers and public health practitioners for improving public health in low- and middle- income countries?*”. These ‘synthetic constructs’ can be viewed as concepts that may provide an insight into some of the complexities of utilising research for action in LMICs. The four concepts identified from the literature were: tension between ‘global’ and ‘local’ evidence; complexities in accessing and creating evidence; contextualising strategies for KT in LMICs; and the unique role of NGOs in the KT process. Although these concepts were drawn from a range of different literature on this topic, there are some unifying themes that enable some generalisations from the findings.

The relationship between knowledge and power, or the ‘politics of knowledge’, was implicit in this literature and influences each concept described above. Power can dictate whose and which forms of knowledge are recognised, valued and used for action, which areas of research are prioritised, and who ‘owns’ knowledge [3, 13, 15] . This is important when considering the value of knowledge in a globalising world shifting from an “industrial economy to a knowledge economy” ([97], p.35). It is known that the value that stakeholders place on knowledge, evidence and research has an impact on KT processes, and can vary considerably across individuals, organisations, systems and sectors [82]. This review suggests that power and politics can influence this.

The significance of partnerships that influence KT, both global-local partnerships, as well as partnerships between researchers and decision-makers, emerged as a theme across all the concepts that were developed during this review. Analysis of certain aspects of such partnerships may therefore be a valuable undertaking in order to maximise the potential for effective KT.

Capacity building focusing on different aspects of KT was another theme that emerged through the concepts – including research capacity; capacity of decision-makers to utilise research evidence; and capacity of various stakeholders to employ KT strategies. It was clear in the literature that the key to effective capacity building in all three of these areas was through institutional strengthening, which should be carried out alongside the capacity building of individuals. This can play a role in building supportive systems and structures for KT in LMICs.

KT has been described as having the ability to redress global inequities [8] through enabling the use of evidence to improve practice and policy. As described in this review, there are still many issues that can hamper effective utilisation of research in LMIC settings. Analysis of these issues, through research or monitoring and evaluation, in order to improve them, is therefore an important factor in improving health and global inequity. Additionally, there is an acknowledged need for further theoretical development to better understand KT in order to improve it [113]. Greenhalgh and Wieringa [114] call for research in the field of KT to “move beyond a narrow focus on the ‘know-do-gap’ to cover a richer agenda” (p. 501). This “richer agenda” may include some of the complexities in LMICs described in this review, including the global-local dynamic, links between power and knowledge, and the contextual factors that influence capabilities in LMICs to access, generate and utilise evidence.

## Conclusion

This review has outlined key concepts that arise in the literature around KT for public health in LMICs. Although there are many ways to look at this complex and dynamic area of public health, this review focuses on the themes of global-local interactions, the value placed on evidence, the contextualisation of KT for LMICs, and the unique role of NGOs in this space. This review utilised the CIS method of inquiry, which allowed a flexible approach to interpreting the literature on the topic. This method was useful in allowing the synthesis of disparate themes within a large body of literature and enabled the development of some theoretical concepts relating to the topic. The result of this analysis is a generalised overview of this huge body of literature. Each concept itself could have been analysed to a much greater depth. However, this analysis has drawn out some key theoretical concepts that may inform practice or further research.

## Additional files

Additional file 1: Search Strategy. Search terms used in original search for articles. (DOCX 27 kb)

Additional file 2: Data Extraction Table. Summary of all articles analysed for the CIS review. (DOCX 51 kb)

Additional file 3: Synthetic Construct Analysis. Articles coded against ‘synthetic constructs’. (DOCX 38 kb)

## Abbreviations

CIS: Critical interpretive synthesis
KT: Knowledge translation
LMIC: Low- and middle- income country
NGO: Non-government organisation
