# Supplementary material for: Knowledge translation for public health in low- and middle- income countries: a critical interpretive synthesis
Source: Glob Health Res Policy. 2018 Oct 22;3:29. doi: 10.1186/s41256-018-0084-9 (PMC6196454; doi:10.1186/s41256-018-0084-9)
Supplement: Supplementary file 1 — Search Strategy. Search terms used in original search for articles. (DOCX 27 kb) [file 41256_2018_84_MOESM1_ESM.docx]

**Additional File 1: Search Strategy**

| Concept | Search String |
| --- | --- |
| Research utilisation | diffusion of innovation/ or translational medical research/  or  (((research or evidence or policy) adj2 (practice or utili* or "use" or utilization or utilisation or uptake or implement* or diffusion or disseminat* or communicat*)) or (Knowledge adj2 (transfer* or translat* or action or exchange)) or know-do gap* or KT or KTA).tw,kw. |
| Low- and middle-income countries  *Based on ISI list of developing countries [1]* | Developing Countries/  or  (((Developing or less developed or low resource or disadvantaged or resource limited or poor or low* income* or low middle income) adj (countr* or region* or nation* or area* or setting*)) or LMIC* or global health).tw,kw.  or  (caribbean or west indies or cuba or dominica or grenada or haiti or jamaica or saint lucia or "saint vincent and the grenadines" or central america or belize or costa rica or el salvador or guatemala or honduras or nicaragua or panama or latin america or mexico or south america or argentina or bolivia or brazil or colombia or ecuador or guyana or paraguay or peru or suriname or venezuela or asia or kazakhstan or kyrgyzstan or tajikistan or turkmenistan or uzbekistan or russia or siberia or cambodia or east timor or indonesia or laos or malaysia or myanmar or philippines or thailand or vietnam or bangladesh or bhutan or india or afghanistan or iraq or jordan or lebanon or syria or turkey or yemen or nepal or pakistan or sri lanka or china or korea or mongolia or albania or bosnia-herzegovina or bulgaria or kosovo or macedonia or moldova or montenegro or belarus or romania or serbia or ukraine or fiji or papua new guinea or vanuatu or micronesia or guam or palau or samoa or tonga or africa or algeria or egypt or libya or morocco or tunisia or cameroon or central african republic or chad or congo or equatorial guinea or gabon or burundi or djibouti or eritrea or ethiopia or kenya or rwanda or somalia or sudan or tanzania or uganda or angola or botswana or lesotho or malawi or mozambique or namibia or south africa or swaziland or zambia or zimbabwe or benin or burkina faso or cape verde or cote d'ivoire or gambia or ghana or guinea or guinea-bissau or liberia or mali or mauritania or niger or nigeria or senegal or sierra leone or togo).tw,kw.  *Country names also searched as MeSH terms.* |

1. International Statistical Institute. List of developing countries 2014 London2014 [15 June 2016]. Available from: <https://www.isi-web.org/index.php/news-items/9-content/8759-dc2014>.
