# Supplementary material for: Knowledge translation for public health in low- and middle- income countries: a critical interpretive synthesis
Source: Glob Health Res Policy. 2018 Oct 22;3:29. doi: 10.1186/s41256-018-0084-9 (PMC6196454; doi:10.1186/s41256-018-0084-9)
Supplement: Supplementary file 2 — Data Extraction Table. Summary of all articles analysed for the CIS review. (DOCX 51 kb) [file 41256_2018_84_MOESM2_ESM.docx]

**Additional File 2: Data Extraction Table**

| **Reference** | **Year** | **Type of paper** | **Methods** | **Summary** | **Key findings** | **Concepts** |
| --- | --- | --- | --- | --- | --- | --- |
| Adrien et al. | 2013 | Primary research | Interviews with project staff in Pakistan and Canada to critically review the HIV/AIDS Surveillance Project in Pakistan over last 8 years. | Role of project in generating and using knowledge; emphasis on two-way capacity building Suggests future opportunities for KT through HASP. | Critically reviews the traditional role of HIC to LMIC KT; describes what needs to happen for good KT to take place; role of NGOs in projects such as these. | Global - local critical analysis. Role of NGOs in KT. |
| Albert et al. | 2007 | Primary research | Interviews with policy makers in Mali about new pharmaceutical guidelines research uptake | Exploring factors influencing research utilisation by policymakers in regard to new pharmaceutical policy. | "These factors include: access to information, relevance of the research, use of research perceived as a time-consuming process, trust in the research, authority of those who presented their view, competency in research methods, priority of research in the policy process, and accountability". (p.2) | Factors affecting policy uptake. |
| Anyaoku & Anunobi | 2014 | Primary research | Citation analysis of articles in a Nigerian journal for HINARI-accessed articles | Articles from a popular Nigerian health journal had citations checked for those that were available from HINARI - a resource that allows free access to several databases for LMICs. | More than 50% of citations were from HINARI accessed journals, although citation analysis has its limitations. | Access to health research for LMICs. |
| Banerji | 2012 | Commentary | History of India's TB program and WHO involvement | Criticism of the WHO for its involvement in India's TB program, which was initially locally-designed, led and based on locally-derived evidence. Later, WHO insisted on a top-down intervention which was not considered appropriate for the local context by some local stakeholders and was not as successful as the locally-led initiative. | WHO's disregard for the locally derived evidence and programming for TB. WHO's simplistic definition of operational research, compared to definitions that underscore the importance of local expertise. | Unequal relationships between global and local players. |
| Bennett et al. | 2015 | Commentary | Discussion of the process and challenges of writing and disseminating a systematic review in Jamaica (or other LMICs) | Describes challenges which are relevant to many LMICs, how these challenges might be met, and examples from Jamaica. Describe a partnership that enables this. | "Challenges were: 1) accessibility to the literature, 2) human resources in research, 3) local funding and 4) knowledge translation (KT)." (p.1095) "Challenges in conducting SR in developing countries can be overcome. Approaches to strengthen KT should be prioritized in order to generate and promote a robust, generalizable evidence base for healthcare and policy." (p.1095) | Systematic Reviews in LMICs. |
| Bissell et al. | 2014 | Primary research | Evaluation of a training program for 36 researchers in Fiji around operational research | Fiji and Pacific OR capacity building courses. Research translation practically non-existent in the region. | Participants learning how to publish papers and present to decision-makers. Early days. | Importance of operational research to knowledge translation. Importance of conforming to Western style publications, etc. |
| Borde et al. | 2014 | Primary research | Literature review, interviews, database consultation | Mapping SDOH research capacities in Brazil | SDOH research mostly carried out by academic community and needs to play a greater role in policy affecting health inequities. | Lack of SDOH research in LMICs |
| Bosi & Gastaldo | 2011 | Commentary | Reflective, critical piece on the different worlds of research, practice and policy in public health | Integration of research, policy and practice is a major challenge due to different actors, paradigms and interests, plus conflict. | There is a need to reflexively examine the connections between research, policy and practice.  Context is all-important (i.e. not one size fits all) | Global-local divide. What is evidence. |
| Brambila et al. | 2007 | Primary research | Review of 44 reproductive health research projects in Guatemala and examination of how they contributed to policy and practice. | Utilisation of research is a gradual process, depends on leadership, collaborative planning, and other factors. Synergistic impact of multiple projects over time need to be considered. | "To maximize the utilization of results, programmes should concentrate on: (1) developing institutional capabilities to use evidence to guide programming and solve problems systematically; and (2) building inter-personal and inter-organizational relationships with those working in both the public and private sectors". (p.234). | Operational research can be very valuable in finding out about the context of certain health issues, which can in turn be valuable to policy and practice. |
| Burchett et al. | 2015 | Primary Research | Interviews with 69 decision makers, researchers and stakeholders in Ghana about their perceived value of public health research and the types of research considered useful in Ghana. | Two traits of research were important: an applied, relevant topic and quickly produced findings. Research which explored implementation issues or identified and increased understanding of health issues was valued. Research around the effectiveness of policies and programmes was not considered as important. | Concepts of research were broad; routine data collection and national data sets were highly valued. Operational research was very highly valued. Distinction between Big Research and Small Research. Explicitly noted importance of qualitative research for understanding context. LMICs have limited ability to compete for funding for big research, so are often stuck with doing the small research. | Broad definitions of what constitutes research. Big vs Small research. Importance of national data sets and operational research. Low perceived value of effectiveness research. |
| Burchett et al. | 2012 | Primary research | Semi-structured interviews in 7 countries about vaccine uptake decision making | There were many variables influencing decision on uptake of vaccines, evidence was just one of them. | Most countries preferred their own evidence as to need for the vaccine. Countries that didn't have research capacity were willing to use other countries. | Importance of local evidence. |
| Cáceres & Mendoza | 2009 | Commentary | Analysis of issues surrounding health research in Peru. | Overview of the local research context in Peru, including factors that relate to global research priorities. | “Research policy development and evaluation processes are poor in Peru, most of the country’s academic research is published in English only, and researchers’ access to funding is limited. Given that the relationship between local academic institutions and foreign research centers is key in developing a ‘‘national science,’’ there is a clear need to reinforce the state’s capacities for management and research oversight and implementation and to encourage the academic community to improve their institutional policies and research frameworks” (p.1792). | Ethics of global research in LMICs Barriers to developing own research priorities |
| Cash-Gibson et al. | 2015 | Commentary | Descriptive | Description of research collaboration between North and South SDH-NET to build capacity in social determinants of health research | "These types of international collaborations can assist in developing solutions to overcome research capacity limitations, further building and strengthening SDH research capacity processes" (p.52). | Institutional capacity strengthening for research. Supportive strategies for SCH research |
| Chen & Yang | 2009 | Commentary | N/A | Access to medical research should be free. Most is not. | Access to research in low income countries is poor. | Access to research databases. |
| Cockcroft et al. | 2011 | Primary research | Interviews and training | Factors affecting ability to uptake research as an elected representative in Botswana | Difficulties – accessing research, human resources for research, IT. Lacked high quality evidence; difficulty interpreting and using it. | Lack of infrastructure for research uptake. |
| Cordero et al. | 2008 | Primary research | Qualitative research with national and international funding agencies on their role in KT in LMICs | How health research funding agencies in LMICs promote KT. | More support from local funding agencies that international for KT. | Need for evaluation of KT. Funding agencies as knowledge brokers. |
| Corluka et al. | 2015 | Primary research | Self-administering online survey to researchers about perceptions of evidence based policymaking in Argentina. | Relatively few links between health research and policymaking. Few opportunities for formalised or informal interactions. | 80% had never been involved in policy, 90% wanted to. Believe policymakers aren't motivated by research and have little understanding of health issues. | Researcher perspectives on policy formulation. |
| Dagenais et al. | 2013 | Primary research | Mixed methods | Analysis of a KT strategy to improve use of research results. | Considerable efforts were invested in KT, however variations occurred between different settings. considerable variation in use was observed from one setting to another. | Process of KT + evaluation |
| Dagenais et al. | 2015 | Primary research | Description of a training program for knowledge brokering in Burkina Faso | Training program aimed to make scientific knowledge more available to knowledge users in Burkina Faso. | Undeveloped research culture requires intensive coaching such as this training program. Knowledge brokers needs to widen their role from translating information to include coaching. | Role for knowledge brokers in LMICs Context very important for KB'ing |
| de-Graft Aikins et al. | 2012 | Primary research | Examination of challenges and opportunities of setting up a North-South research partnership addressing chronic disease in Africa | Describes the partnership in terms of established partnership frameworks. Had four aims - access funding for chronic disease research projects; KT to policy makers; postgraduate education; create a platform for research utilisation. Some of these were more successful than others. Funding was a problem. | Enablers: trust, respect, shared commitment. Barriers: funding, admin, M&E framework. | LMICs prioritise infectious diseases. Need sustainable partnerships for NCD research for capacity building. |
| Delisle et al. | 2004 | Review | Reviewed literature on the role of NGOs in global research | Documentation of the role that NGOs have played in global health research. | NGOs have traditionally played certain roles within research, that could be expanded under the term 'global research' under the right conditions. | Little funding and support for research in LMICs. Global vs local research.  Key role for NGOs in global research. |
| Drake et al. | 2010 | Review | Describes 3 case studies where NGOs have been involved in using evidence to shape evidence informed programs and policies. | Summary of the role of NGOs in translating science to programs. The three case studies show how NGOs can harness their advantages. | NGOs can use and expand on research in public health in order to help develop programmes. NGOs are well-positioned to do this - geographic reach, relationships with stakeholders, advocacy ability for health challenges. | Unique position of NGOs to carry out KT. |
| D'Souza & Sadana | 2006 | Review | Reviewed case studies about health systems research in LMICs | Described challenges and constraints to health research systems in LMICs through analysing case studies. Demonstrated strengths of case studies. | Challenges included lack of coordination, participation, demand for research, accessibility. Constraints were financial capability, human capacity, institutional capacity. | Case studies valuable source of information. Barriers to research in LMICs. |
| El-Jardali et al. | 2014 | Primary research | Structured reflection, interviews, document reviews about knowledge transfer platforms in LMICs | Structured reflection on KT platforms for policy development in LMICs | KTPs have contributed to increasing the awareness of evidence based practice. Required support from policymakers. Sustainability was a challenge. | Evaluation of knowledge translation platforms needs to occur. |
| El-Jardali et al. | 2010 | Primary research | Interviews with policymakers and researchers in Nth Africa and Middle East region about research into policy | Bringing researchers and policymakers together for priority research areas for health systems and policy formulation. | Top five research priorities identified - focusing on these areas will assist with reaching the MDGs. | Health systems research in LMICs. |
| Fabic et al. | 2012 | Review | Systematic review | Review of published papers making use of demographic and health surveys in 84 countries, provided free access by WHO | “Researchers are using DHS data more and more, and as a result health programme managers and policy makers have increasing access to vital health information”(p.604). | Availability of national demographic health surveys has allowed production of more research using local data. |
| Frenk & Chen | 2011 | Commentary | Summary of a symposium on global health research | Global is local. | Strengthening essential health research and mobilising health research partnerships. | Capacity strengthening.  Health systems research. |
| Gadsby | 2011 | Primary research | Document reviews and interviews with donor organisations involved with research capacity strengthening. | Gives an overview of Research Capacity Strengthening in LMICs, models and frameworks and then details about donors in LMICs | Still a very early field. Lots of donor interest and they try to tie it in to their work. | Frameworks for research capacity strengthening. |
| Glover et al. | 2006 | Commentary | Review of Bhutan's access to health information and research | Bhutan has limited access to research information however internet access has allowed better use of the WHO and HINARI free articles. | Geographic and financial barriers to accessing health information. Internet is integral. WHO and HINARI provide access. Great importance placed on traditional medicine research. | Limited access to articles other than what is available through WHO and HINARI. |
| Goyet et al. | 2015 | Review | Systematic review | Research published by Cambodian authors between 2002-12 and how the research topics related to public health priorities | Global priorities for research are funded by global partnerships. NCDs are a priority for the country, but is the focus of only 7% of the research. A health research agenda would help to align priorities. | Research funding for global priorities rather than local Less research on NCDs |
| Hamel & Schrecker | 2011 | Primary research | Case study of Burkina Faso Public health association | Case study on the public health association of Burkina Faso focusing on factors that affect KT strategies in LMICs by these types of organisations. | Importance of having an organisational perspective on KT. | Role of NGOs in KT.  Institutional capacity strengthening. |
| Harris et al. | 2015 | Primary research | Web based survey asking public health editors to rate abstracts from LMIC compared to HIC (blind) | There was a difference in 1 paper where the LMIC author was rated lower than HIC for the same abstract. | “All things being equal, in one of the four abstracts, the respondents were influenced by a high-income source in their rating of research abstracts” (p.1). | Research valued differently depending on source country. |
| Hate et al. | 2015 | Primary research | Interviews with stakeholders associated with an NGO in India, about data sharing. | Data sharing in India amongst researchers and NGOs - describes researchers’ thoughts and hesitations in sharing data. | Three main themes: concern about acknowledgement of research; scepticism about sharing data; data sharing environment complex and confusing. | Data sharing - implications for access to data. |
| Hawkes | 2012 | Commentary | N/A | Elselvier providing open access to some journals to LMICs. | 15% of the content of a journal can be downloaded for free through a special portal. Similar to other projects such as HINARI. | Access to research |
| Hawkes et al | 2015 | Primary research | Evaluate experience of capacity building training for policy makers (individuals, organisations, institutions) to increase the level of evidence use in policy making in 5 countries: India, Bangladesh, Gambia, Nigeria. | Different barriers and enablers existed in each of the 5 countries in terms of evidence utilisation. | Capacity building for individuals in evidence utilisation is not enough to ensure evidence use in policymaking is sustainable. The politics of the processes need to be understood. | Understanding between researchers and policymakers. What is regarded as evidence? |
| Hennink & Stephenson | 2005 | Primary research | Interviews with researchers, policy makers and practitioners at the local and national level. | Lack of appreciation of research to policy and programmes. Lack of appropriate packaging of evidence. | Overcoming barriers requires effort. Donor presence has a big impact. Increased collaboration needed. | Lots of barriers between researchers and policy makers. |
| Hyder et al. | 2010 | Primary research | Semi-structured in-depth interviews in 7 LMICs | Perspectives of policymakers about the use and impact of research. | Lists of findings and discussion around how people perceive evidence. | Suggestions for both policymakers and researchers.  Perceptions on evidence. |
| Jao et al. | 2015 | Primary research | Interviews with researchers about data sharing in Africa | Hesitations around data sharing showing need to build trust in the research field. | Complex and new topic. Concerns about who shares data and for what. Risks to study participants and researchers. | Ethics of data sharing. Other researchers using hard-earned data. Issues to consider in generating local research. |
| Jönsson et al. | 2007 | Primary research | Surveys and interviews with researchers in Lao PDR regarding uptake of research into policy | Pessimism about overcoming barriers to getting research evidence into policy. Inability to influence policy process. Lack of continuous capacity building. | Awareness of the barriers and need for evidence use in health policy making. Donor-led research may set the agenda, and may mean there is not much relevance for the local context. | Global to local Contextual barriers of a LMIC |
| Kasonde & Campbell | 2012 | Commentary |  | Knowledge transfer platform in Zambia. Describes 9 key points for success. | KT platforms provide leadership and cohesion for KT. | KTPs can be very useful platforms. Could be located in NGO or govt |
| Kendall & Langer | 2015 | Primary research | Interviewed 25 key experts and researchers in the field of maternal and child health about existing gaps in LMICs post 2015 | Still lots of gaps and opportunities for knowledge generation and translation. | Need for health systems research; Attitudes, behaviour and power relationships important factors; NCDs, urbanisation and health inequities important areas for research and KT. | Health systems research, implementation research. Noted gap about burgeoning of NCDs. |
| Keusch et al. | 2010 | Commentary | Summarises changes in global malaria research and action over the last century | Malaria eradication research and action through partnerships with institutions in LMICs. A global strategy with local linkages. | Global research networks have contributed to action on malaria eradication. Research needs to be done at the local level for good implementation. | Global-local partnerships. |
| Lavis et al. | 2010 | Primary research | Survey of researchers in 10 LMICs about involvement in collaborative bridging activities to increase uptake of research into policy | Studied efforts to bridge the gap between research, policy and practice. | Future initiatives could support bridging activities. | Knowledge networks. |
| Mahendradhata et al. | 2014 | Description of program | Describes 10 years of operational research in Indonesia | Reiterates importance of local ownership of OR, and that most is still funded and overseen by international agencies. | OR still being funded and implemented by international agencies - need for it to become more local. Demonstrated effectiveness of OR and OR training in Indonesia - needs support with publications, English writing, basic research skills, etc. | Operational research |
| Mbonye & Magnussen | 2013 | Description of program | Describes a training program and partnership to promote research into practice and policy in Uganda | Team was built to discuss strategies for overcoming significant health problems in Uganda. | Details slow pace of research into policy in Uganda (e.g. Certain drugs to be used/not used). Partnerships needs to be strengthened with key people from government. Discusses need for self-motivated individuals, adequate funding, stable internet. | Barriers to evidence uptake. |
| Mijumbi et al. | 2014 | Primary research | Case study detailing rapid response service for policymakers in Uganda. | Rapid response was a feasible strategy in response to urgent needs - increases confidence of policymakers. | Rapid response mechanisms are feasible in LMICs. | Rapid response. Policymakers views. |
| Miranda & Zaman | 2010 | Commentary | N/A | Translating global research to local contexts in developing countries does not always work. Innovative solutions need to be developed to ensure that research is relevant to local contexts. | Research community from LMICs should have major saying shaping research and interventions, as context is crucial. | Global-local. Importance of context. |
| Moat et al. | 2014 | Primary research | Surveys | Evaluating the effectiveness of evidence briefs and deliberative dialogues in LMICs | Evidence briefs and deliberative dialogues are highly regarded and are linked with intention to act. | Effectiveness of evidence briefs and deliberative dialogues |
| Mori et al. | 2014 | Primary research | Interviews and document reviews. | Describes the process of updating the Standard Treatment Guidelines and National Essential Medicine List in Tanzania - examines evidence use in this process. | Limited use of evidence was used in making these decisions - the main factor in making the decisions were discretionary judgement and experience. Risks ineffective and expensive policy decisions. | What is evidence and how do decision makers use it. |
| Olivier | 2016 | Review | Reviewed literature to find benefits and challenges to NGO-R partnerships, and approaches that promote successful partnerships | International partners are a key feature of NGO-R partnerships, which creates some ethical challenges. Possible imbalance in relationships. Describes positive role of NGOs in research uptake. Benefits, challenges, strategies to sustain partnerships. | Benefits: mutual learning; complementary expertise; improved KT; increased access to communities and govt. reps - can adapt research to local communities. Challenges: asymmetrical power relations; divergence in goals and approaches - partnership tensions, lost in translation; lack of recognition for partner contribution.  Strategies: improving communication; transparency in decision-making; mutual respect and reciprocity; developing trust. | Global-local power imbalance. Role of NGOs. |
| Onwujekwe et al. | 2015 | Primary research | Interviews and focus groups with policy makers in Sth Africa and Cameroon | Value of research in health policy formation, what affects it, and value of partnership with researchers | Research plays a role in policy but not as a key driver. | Partnerships important. Research not huge driver of policy. |
| Orem et al. | 2012 | Primary research | Literature review and key informant interviews | Stakeholders perspectives on KT in policy in Uganda. | Literature review explores main facilitating factors for KT in LMICs. Interviews were compared to this. Role of CSOs in KT is largely unexplored. | Facilitating factors for KT in LMICs. NGOs. |
| Orem et al. | 2014 | Primary research | Interviews with policymakers and researchers in Uganda | Explores evidence uptake in public health policy in Uganda. | Evidence utilisation does not happen as much as it should. Lack of clarity about KT and evidence in general. Need to understand which KT tool to use in which setting. | Context for KT tools. Lack of knowledge about KT and evidence. |
| Orem et al. | 2013 | Primary research | Interviews with stakeholders about KT in health policy in Uganda. | Links and platforms for KT need to be built. Capacity strengthening. Role of CSOs in KT. | CSOs and media are potential KT players. | Role of NGOs. |
| Orem et al. | 2014b | Primary research | Interviews with key informants. | How evidence uptake was supported or not by stakeholders involved in the changing of malaria policy in Uganda. Stakeholder analysis was used. | Stakeholders played various roles with different level of support for evidence use. | Values of evidence.  Role of CSOs and other NGOs discussed. |
| Orem et al. | 2014c | Primary research | Case study and mixed methods | Perceptions on use of evidence use to change malaria policy - including access to evidence, uptake, barriers and facilitators. | Evidence was used but inconsistently. Availability of high quality evidence, institutional capacity, networks plus other factors were important. | What is evidence. E.g. D drug efficacy evidence was used but not community acceptance evidence. |
| Quaglio et al. | 2014 | Commentary | N/A | The EU should support operational research in LMICs | Many LMICs are rich in data but it is under-used. OR can assist with ensuring that research is utilised. | Operational Research & pilot projects |
| Reddy & Sahay | 2016 | Primary research | Interviews with decision-makers in India around research regarding HIV/TB co-infection. | Describes views of decision-makers in terms of evidence that they trust and act on in relation to TB/HIV co-infection, and generally. | Decision-makers valued context-specific (i.e. Country or region specific evidence) over global evidence. Have to trust researchers in lieu of not being able to assess evidence themselves. Local evidence based in the right context is ultimately more important. Trust in the researcher. Need to have research presented in terms that are relevant to decision-makers. | Local contextual evidence important.  Global-local. |
| Redman-MacLaren et al. | 2010 | Primary research | Literature review and focus groups with researchers and health care workers. | Literature review found historical inequity in conducting and accessing health research. Local researchers now working towards setting priorities in Solomon Islands. | Action from the literature search has influenced nature of research at one S.I. institution. Need to strengthen capacity of local researchers. | Historical inequities in research. Who chooses the research agenda. Capacity strengthening. |
| Rodríguez et al. | 2015 | Primary research | Retrospective case study using document review and interviews in 3 countries. | Explores the use of evidence in policy formulation for iCCM in 3 countries. | Policy formulation was influenced by a range of evidence including research, experiences, and global and local pilot projects. Highlights need for local data to answer key questions from policymakers. | Global-local. Importance of context.  What is evidence. |
| Rosenbaum et al. | 2011 | Primary research | User tests of evidence summaries in 6 countries followed by interviews. | Views of what features of evidence summaries were useful for policymakers in 6 countries. | Policy makers’ preference for local knowledge.  Difficulty of policymakers in comprehending research language. | Barriers to policy formulation. Evidence summaries as KT tool. |
| Ruggeri | 2014 | Commentary | N/A | Journal partnerships can help to alleviate some of the issues preventing research dissemination in sub-Saharan Africa. | Describes some of the common issues with researchers disseminating their findings in LMICs, and how journal partnerships can alleviate these. Journal partnerships as a KT tool. | Operational Research. Research partnerships. |
| Siminerio & Mbanya | 2011 | Description of project | N/A | Describes a partnership project to fund KT projects in LMICs. | Commitment and action still needed at a high level. Translational research can assist this process. | Non-communicable disease research Determining research priorities Role of NGOs |
| Siron et al. | 2015 | Review | Scoping review of literature | KT strategies used in low income countries. | Review of articles evaluating KT strategies in low income countries. Great diversity of tools used, and were context specific. Recommendation to more closely examine the context characteristics. Lack of evaluation for KT noted. | Lack of evaluation for KT. KT strategies used. |
| Ssengooba et al. | 2011 | Primary research | A case study and interviews. | Analysed research - policy discourses amongst key stakeholders in two HIV prevention research projects. | Shared platforms, pilots, operations research were key facilitators. Nature of evidence was weighed differently by different stakeholders. | What is evidence. Barriers and facilitators to evidence use. |
| Storeng & Behague | 2014 | Primary research | Interviews with Safe Motherhood Initiative stakeholders about Evidence Based Advocacy | Evidence based advocacy - new priority and tactic of the initiative. Move from a rights based advocacy approach to "playing the numbers game". | "Many stakeholders feel an ambivalence about having to provide hard evidence for safe motherhood initiatives instead of more holistic, feminist, human rights based advocacy. The focus on SDH does help to frame arguments with an equity focus". P.260. | What is evidence? What kind of evidence changes policy? |
| Sullivan et al. | 2015 | Commentary | N/A | Definition of Knowledge Management and relation to global health, with an example of a USAID project in Bangladesh | There are specific strategies to KM. This article was mostly focused on global agencies and development practitioners, and gave the example of e-learning. | Social knowledge management. |
| Sumner et al. | 2011 | Review | Overview of research into policy; in relation to sexual and reproductive health | Develops a framework for understanding research uptake in SRH health policy processes in LMICs. | Overview of research into policy and its history. Context important, e.g. Sexual health politics. Description of what is evidence. | What is evidence? |
| Tharyan | 2010 | Commentary | Brief overview of the situation of development and access to research in LMICs | Getting the right sort of research; getting evidence used; research into policy | LMICs can be at risk if research is not carried out within local context. There is still a long way to go with access to research as well. | Access to research in low income countries is poor.  Global to local. |
| Tomson et al. | 2005 | Primary research | Self-administered questionnaire about research in policy making with 90 decision makers at a conference in Lao PDR | Factors inﬂuencing decision-makers’ perceptions of usefulness of research. Relationships important. More research to do on interface between policy and research. | Policy makers that were aware of previous research in the area were more likely to use it. Decision makers were not sure what 'research' actually is. | What is evidence? |
| Uthman et al. | 2015 | Primary research | Quantitative - compared articles published with country-level indicators from the World Bank for each country of origin. | Calculated amount of research produced in Africa from 2000-15, as well as an analysis of the factors associated with research output. | Improvement in health research in the region over this time, however needs the way forward needs to be mapped. More research was associated with economic growth. | Link of economic growth with research output. |
| Valinejadi et al. | 2016 | Primary research | Mixed methods - survey with diabetes researchers in Iran. | Evaluating KT strategies for diabetes research in Iran | Low status of diabetes KT in Iran. Challenges at organisational and macro level. KT should have more of a function in the diabetes research system. | KT for NCDs. |
| Walugembe et al. | 2015 | Primary research | Case study with researchers about research utilisation by policymaking process. | Most researchers said their study findings were used to influence policymaking processes, but not sure how. | Stakeholder engagement critical through multiple channels of communication is critical, and paying attention to context. | Difficult to know how much impact research has on policy outcomes. |
| Waqa et al. | 2013 | Primary research | Evaluated knowledge brokering process in Fiji | Description of a knowledge exchange program amongst high-level NGO and government organisation key staff and researchers in Fiji around the topic of obesity. | This type of capacity building program can be tailored to other LMICs. | Knowledge brokering |
| Welch et al. | 2009 | Review |  | Describes inequalities in health and how KT in different areas can reduce inequalities | Focusing KT strategies on areas that will reduce inequalities, e.g. Water and sanitation. | Overview of how KT can be targeted towards interventions that reduce inequalities both in and between countries. |
| Winnik et al. | 2013 | Primary research | Analysis of journal submissions and publications and citations with GDP of a country | Compared journal submissions/publications/citations and mapped against GDP of countries, concluding that research from lower GDP countries were less likely to be published and cited. | Possible reasons for this included the wealth of nations may be a marker of other factors affecting scientific success; research from non-wealthy nations may appear less relevant; established peer networks of researchers may affect the ratings. | Country of origin GDP compared to journal submissions, publications and citations. |
| Woelk et al. | 2009 | Primary research | Case studies | Compared evidence use between two policy development processes - magnesium for eclampsia and bed nets for malaria. Found differences particularly related to the "evidence". | "Evidence" was seen as different between the two conditions. Local champions were important. Policy communities were evident in eclampsia. There were more players in the malaria one. KT often dependent on a few key people. Context important. | What is evidence. KT is complex and sensitive process. |
| Yazdizadeh et al. | 2014 | Primary research | Surveys and quantitative. Network analysis. | Analysis of knowledge networks in Iran. Existing networks did not seem to be very effective. Suggestions for strategies for strengthening and evaluating research networks. | Management and governance problems need to be resolved in order to strengthen networks. | Strengthening knowledge networks in LMICs. |
| Yehia & El Jardali | 2015 | Primary research | Intervention followed by semi-structured interviews with 10 policymakers and key informants | After 6 months, improvements in relationships and KT actions. | Use of KT in Lebanon promising. Utilisation of KT platforms. Framing of the problem and context was critical. | KT platforms |
| Young | 2005 | Primary research | Stakeholder workshops in LMICs about research into policy | Some of the important factors are political context, access to research, external interference and the emergence of civil society as a key player. The research-policy setting has not been studied much in LMICs. | Two strategies that have found to be useful were think tanks and networking. | Interference from donors on research and policy agenda. Lack of access to research. Role of NGOs. Think tanks and networking. |
| Yousefi-Nooraie et al. | 2009 | Primary research | Survey of participants in a systematic review workshop | Importance of systematic reviews for decision making in LMICs. Participant views on access to and production of systematic views in LMICs | Identification of important features that contribute to conducting and using systematic reviews in LMICs. | Systematic Reviews in LMICs. |
| Zachariah & Draquez | 2012 | Commentary | N/A | Importance of OR in NGOs | What works in NGOs; What's happening with MSF; Gap in NGOs. Operational research is not a luxury; it should be an integral part of all NGOs’ programmes. | Role of NGOs. Gap in NGOs. Importance and role of OR. |
| Zachariah et al. | 2010 | Review |  | NGOs as researchers - lists the benefits and challenges.  Defines operational research from an NGO point of view. | NGO involvement in research creates many benefits for NGOs, KT and the community. | Health research can make NGOs better advocates by adding a strong evidence base. They can be directly involved in the translation of evidence. |
| Zachariah et al. | 2012 | Commentary | N/A | Features of operational research that lead to successful translation into policy and practice in LMICs.  Definitions of operational research. | Defines barriers and solutions to doing research in LMICs, disseminating and translation of findings. | Role of NGOs. Access to research. Importance and role of OR. |
| Zachariah et al. | 2014 | Primary research | Retrospective cohort study (survey) | Describes implementation of an Operational Research training course held with participants from LMICs. Survey of resulting publications and changes to policy and practice. | OR can foster rapid translation of research findings into policy and practice. | Importance of OR. |
