# Supplementary material for: Knowledge translation for public health in low- and middle- income countries: a critical interpretive synthesis
Source: Glob Health Res Policy. 2018 Oct 22;3:29. doi: 10.1186/s41256-018-0084-9 (PMC6196454; doi:10.1186/s41256-018-0084-9)
Supplement: Supplementary file 3 — Synthetic Construct Analysis. Articles coded against ‘synthetic constructs’. (DOCX 38 kb) [file 41256_2018_84_MOESM3_ESM.docx]

**Additional File 3: Synthetic Construct Analysis**

| Synthetic Construct | Topics included | References | Concept Papers – Purposively sampled |
| --- | --- | --- | --- |
| Tension between global and local | Complex relationships between global and local public health | Keusch (2010)  Frank & Chen (2011)  Kendall & Langer (2015) | Sanders et al. (2004)  McCoy et al. (2004)  World Health Organisation (2008) |
|  | Partnerships between HICs and LMICs | Cash-Gibson et al. (2015)  De-Graft Aikins et al. (2012) |  |
|  | Unequal relationships | Banerji (2012)  Cacarez & Mendoza (2009)  Young (2005) |  |
|  | Global research vs local research | Cordero et al. (2008)  Goyet et al. (2015)  Mahendradhata (2014)  Harris et al. (2015) |  |
|  | From one setting to another | Adrien et al. (2013)  Burchett et al. (2012)  Burchett et al. (2015) |  |
|  | Who sets/what shapes the research agenda - power | Cacarez & Mendoza (2009)  Jonsson et al. (2007)  Redman-MacLaren (2010)  Bissell et al. (2014) |  |
|  | Two-way learning | Adrien et al. (2013) |  |
|  | What is evidence and knowledge in public health? | Cacarez & Mendoza (2009)  Orem et al. (2014)  Young (2005) | La Paige (2010)  Armstrong et al. (2014)  Greenhalgh & Wieringa (2011) |
|  | Many different types of evidence used in policy making and practice | Reddy & Sahay (2016)  Rodriguez et al. (2015)  Dagenais et al. (2013)  Hawkes et al. (2015)  Hyder et al. (2010)  Mori et al. (2014)  Orem et al. (2014)  Onwujekwe et al. (2015)  Storeng & Behauge (2015)  Burchett et al. (2015) |  |
|  | Use of qualitative evidence | Bosi & Gastaldo (2011)  Burchett et al. (2015) |  |
|  | Nature of evidence weighed differently by different players | Rodriguez et al. (2015)  Ssengooba et al. (2011)  Sumner et al. (2011) |  |
|  | Limited understandings of what research is | Hate et al. (2015)  Bissell et al. (2014)  Reddy & Sahay (2016)  Orem et al. (2014)  Tomson et al. (2005) |  |
|  | Big research vs small research | Cacarez & Mendoza (2009)  Jonsson et al. (2007)  Burchett et al. (2015) |  |
|  | Evidence based advocacy – numbers game | Storeng & Behague (2014) |  |
|  | Published research – link to health needs? | Goyet et al. (2015) |  |
|  | Data sharing and trust, powerlessness | Hate et al. (2015)  Uthman et al. (2015)  Jao et al. (2015) |  |
|  | Health systems research | Jonsson et al. (2007)  Kendall & Langer (2015) |  |
| Creating and Accessing Evidence: “data rich, information poor” | Data gathering, including big data sets – “data rich, information poor” | Fabic et al. (2012)  Bissell et al. (2014)  Hawkes et al. (2015)  Burchett et al. (2015) | Beaglehole et al. (2011) |
|  | Operational Research and pilot projects | Ruggeri (2014)  Bissell et al. (2014)  Brambila et al. (2007)  Mahendradhata (2014)  Quaglio (2014)  Zachariah et al. (2010); (2012); (2014) |  |
|  | Barriers to conducting and disseminating research | Winnik et al. (2013)  Uthman et al. (2015)  Orem et al. (2014)  Ssengooba et al. (2011)  Valinejadi et al. (2016) |  |
|  | Linking research to action | Lavis (2010)  Walugembe et al. (2015) |  |
|  | Access to research, eg. HINARI | Anyaoku et al. (2014)  Chen & Yang (2009)  Goyet et al. (2015)  Glover (2006)  Tharyan (2010) |  |
|  | Skills for researchers and policymakers | Brambila et al. (2007) |  |
|  | Research partnerships | Ruggeri (2014)  Zachariah et al. (2012)  Kendall & Langer (2015)  Brambila et al. (2007)  Olivier et al. (2016) |  |
|  | Strengthening organisational capacity (researchers and policymakers) | Young (2005)  Hamel & Shrecker (2010)  Gadsby (2011)  Brambila et al. (2007)  Goyet et al. (2015)  D’Souza & Sadana (2005) |  |
|  | NCD research/determining research priorities | Kendall & Langer (2015)  Valinejadi et al. (2016)  Siminero & Mbenya (2011)  De-Graft Aikins et al. (2012) |  |
| KT strategies need to be contextualised for LMICs | Barriers to policy formulation | Hennink & Stevenson (2005)  Mbonye & Magnussen (2013)  Walengumbe et al. (2013)  Burchett et al. (2012)  Cockcroft et al. (2011)  Jonsson et al. (2007) | Dagenais et al. (2009)  Hanney et al. (2003)  La Paige (2010)  LaRocca et al. (2012)  Welch et al. (2013) |
|  | Policymaker and researcher views of research uptake | Hawkes et al. (2015)  Hennink & Stevenson (2005)  Mijumbi et al. (2014)  Albert et al. (2007)  Hyder et al. (2010)  Corluka et al. (2015) |  |
|  | Dissemination – by donor agencies and NGOs | Hennink & Stevenson (2005)  Tomson et al. (2005) |  |
|  | Evaluating KT strategies – gap | Cordero et al. (2008)  Hamel & Shrecker (2010)  Dagenais et al. (2013)  Siron et al. (2015)  Yehia & El-Jardali (2015)  El-Jardali et al. (2010); (2014) |  |
|  | Knowledge brokers | Dagenais et al. (2015)  Waqa et al. (2013); |  |
|  | Knowledge translation platforms/ formal knowledge networks | Kasonde & Campbell (2012)  El-Jardali et al. (2014)  Yazdizadeh et al. (2014)  Lavis (2010)  Orem et al. (2012) |  |
|  | Feasibility of some KT techniques and frameworks | Mijumbi et al. (2014)  Moat et al. (2014)  Orem et al. (2012)  Rosenbaum et al. (2011)  Yehia & El-Jardali (2015)  Young (2005) |  |
|  | Relationships between researchers and policymakers | Valinejadi et al. (2016)  Ssengooba et al. (2011)  Bosi & Gastaldo (2011)  Hawkes et al. (2015)  Reddy & Sahay (2016) |  |
|  | Analysing the impact of research | Valinejadi et al. (2016)  Sumner et al. (2011)  El-Jardali et al. (2014) |  |
|  | KT to redress global inequities | Kendall & Langer (2015)  Welch et al. (2009) |  |
|  | Social Knowledge Management | Sullivan et al. (2015) |  |
|  | Need for structures, processes, stability, relationships | Hawkes et al. (2015)  Orem et al. (2012)  Orem et al. (2014) |  |
|  | Increasing role of civil society in KT | Kendall & Langer (2015)  Orem et al. (2012)  Young (2005) |  |
|  | Funding agencies as players in KT | Cordero et al. (2008) |  |
| NGOs have a unique position in KT processes | As implementing partners | Zachariah et al. (2012)  Zachariah (2010)  Adrien et al. (2013)  Desisle et al. (2015)  Young (2005) | Kothari & Armstrong (2011)  Wilson et al. (2010) |
|  | In KT strategies | Orem et al. (2012)  Hamel & Shrecker (2010)  Orem et al. (2013)  Orem et al. (2012) |  |
|  | NGO + researcher partnerships | Olivier et al. (2016) |  |
|  | Advocacy | Dagenais et al. (2013)  Woelk et al. (2009) |  |
|  | In research | Drake et al. (2010)  Orem et al. (2014) |  |
